# SARS-CoV-2 infection in immunosuppression evolves sub-lineages which independently accumulate neutralization escape mutations

**DOI:** 10.1093/ve/vead075

**Published:** 2023-12-28

**Authors:** Gila Lustig, Yashica Ganga, Hylton E Rodel, Houriiyah Tegally, Afrah Khairallah, Laurelle Jackson, Sandile Cele, Khadija Khan, Zesuliwe Jule, Kajal Reedoy, Farina Karim, Mallory Bernstein, Thumbi Ndung’u, Mahomed-Yunus S Moosa, Derseree Archary, Tulio de Oliveira, Richard Lessells, Richard A Neher, Salim S Abdool Karim, Alex Sigal

**Affiliations:** Centre for the AIDS Programme of Research in South Africa, 719 Umbilo Road, Durban 4001, South Africa; Africa Health Research Institute, 719 Umbilo Road, Durban 4001, South Africa; Africa Health Research Institute, 719 Umbilo Road, Durban 4001, South Africa; Division of Infection and Immunity, University College London, UCL Cruciform Building Gower Street, London WC1E 6BT, UK; KwaZulu-Natal Research Innovation and Sequencing Platform, 719 Umbilo Road, Durban 4001, South Africa; Centre for Epidemic Response and Innovation, School of Data Science and Computational Thinking, Stellenbosch University, Francie Van Zijl Drive, Cape Town 7505, South Africa; Africa Health Research Institute, 719 Umbilo Road, Durban 4001, South Africa; Africa Health Research Institute, 719 Umbilo Road, Durban 4001, South Africa; Africa Health Research Institute, 719 Umbilo Road, Durban 4001, South Africa; School of Laboratory Medicine and Medical Sciences, University of KwaZulu-Natal, 719 Umbilo Road, Durban 4001, South Africa; Africa Health Research Institute, 719 Umbilo Road, Durban 4001, South Africa; School of Laboratory Medicine and Medical Sciences, University of KwaZulu-Natal, 719 Umbilo Road, Durban 4001, South Africa; Africa Health Research Institute, 719 Umbilo Road, Durban 4001, South Africa; Africa Health Research Institute, 719 Umbilo Road, Durban 4001, South Africa; Africa Health Research Institute, 719 Umbilo Road, Durban 4001, South Africa; School of Laboratory Medicine and Medical Sciences, University of KwaZulu-Natal, 719 Umbilo Road, Durban 4001, South Africa; Africa Health Research Institute, 719 Umbilo Road, Durban 4001, South Africa; Africa Health Research Institute, 719 Umbilo Road, Durban 4001, South Africa; Division of Infection and Immunity, University College London, UCL Cruciform Building Gower Street, London WC1E 6BT, UK; School of Laboratory Medicine and Medical Sciences, University of KwaZulu-Natal, 719 Umbilo Road, Durban 4001, South Africa; HIV Pathogenesis Programme, University of KwaZulu-Natal, 719 Umbilo Road, Durban 4001, South Africa; Ragon Institute of MGH, MIT and Harvard University, 400 Technology Square, Cambridge, MA 02139, USA; Department of Infectious Diseases, Nelson R. Mandela School of Clinical Medicine, University of KwaZulu-Natal, 719 Umbilo Road, Durban 4001, South Africa; Centre for the AIDS Programme of Research in South Africa, 719 Umbilo Road, Durban 4001, South Africa; KwaZulu-Natal Research Innovation and Sequencing Platform, 719 Umbilo Road, Durban 4001, South Africa; Centre for Epidemic Response and Innovation, School of Data Science and Computational Thinking, Stellenbosch University, Francie Van Zijl Drive, Cape Town 7505, South Africa; Department of Global Health, University of Washington, 3980 15th Avenue NE, Seattle, WA 98105, USA; KwaZulu-Natal Research Innovation and Sequencing Platform, 719 Umbilo Road, Durban 4001, South Africa; SIB Swiss Institute of Bioinformatics, Quartier Sorge - Bâtiment Amphipôle, Lausanne 1015, Switzerland; Biozentrum, University of Basel, Spitalstrasse 41 4056, Basel, Switzerland; Centre for the AIDS Programme of Research in South Africa, 719 Umbilo Road, Durban 4001, South Africa; Department of Epidemiology, Mailman School of Public Health, Columbia University, 722 West 168th Street, New York, NY 10032, United States; Centre for the AIDS Programme of Research in South Africa, 719 Umbilo Road, Durban 4001, South Africa; Africa Health Research Institute, 719 Umbilo Road, Durban 4001, South Africa; School of Laboratory Medicine and Medical Sciences, University of KwaZulu-Natal, 719 Umbilo Road, Durban 4001, South Africa

**Keywords:** SARS-CoV-2 evolution, advanced HIV disease, immunosuppression, prolonged infection, variants of concern

## Abstract

One mechanism of variant formation may be evolution during long-term infection in immunosuppressed people. To understand the viral phenotypes evolved during such infection, we tested SARS-CoV-2 viruses evolved from an ancestral B.1 lineage infection lasting over 190 days post-diagnosis in an advanced HIV disease immunosuppressed individual. Sequence and phylogenetic analysis showed two evolving sub-lineages, with the second sub-lineage replacing the first sub-lineage in a seeming evolutionary sweep. Each sub-lineage independently evolved escape from neutralizing antibodies. The most evolved virus for the first sub-lineage (isolated day 34) and the second sub-lineage (isolated day 190) showed similar escape from ancestral SARS-CoV-2 and Delta-variant infection elicited neutralizing immunity despite having no spike mutations in common relative to the B.1 lineage. The day 190 isolate also evolved higher cell–cell fusion and faster viral replication and caused more cell death relative to virus isolated soon after diagnosis, though cell death was similar to day 34 first sub-lineage virus. These data show that SARS-CoV-2 strains in prolonged infection in a single individual can follow independent evolutionary trajectories which lead to neutralization escape and other changes in viral properties.

## Introduction

Current data supports the notion that SARS-CoV-2 variants arise without intermediate forms circulating in the population. Mechanisms of variant formation which are consistent with such sudden emergence include infection of and evolution in an animal reservoir (Bayarri-Olmos et al. 2021; Fenollar et al. 2021; Griffin et al. 2021; Hoffmann et al. 2021; Meekins, Gaudreault, and Richt 2021; Ren et al. 2021; Hale et al. 2022; Koeppel et al. 2022; Kuchipudi et al. 2022; Zhou et al. 2022) or evolution in long-term infection in one or possibly a small group of immunosuppressed people (Avanzato et al. 2020; Choi et al. 2020; Baang et al. 2021; Corey et al. 2021; Hoffman et al. 2021; Jensen et al. 2021; Kemp et al. 2021; Peacock et al. 2021; Karim et al. 2021b; Cele et al. 2022; Maponga et al. 2022; Riddell et al. 2022; Wilkinson et al. 2022). This would be followed by spread of the evolved virus back to the general population. Analysis of multiple long-term infections in immunosuppressed people has demonstrated recurrent mutations that are associated with escape from neutralizing antibodies (Wilkinson et al. 2022). However, mutations outside of the spike glycoprotein are also common, although for most, the effect on virus phenotype is not well understood (Kistler, Huddleston, and Bedford 2022).

Among the causes of immunosuppression is advanced HIV disease (Hoffman et al. 2021; Karim et al. 2021b; Cele et al. 2022; Riddell et al. 2022), defined as a CD4 T-cell count < 200 cells/µl. There are approximately 8.5 million people living with HIV in South Africa alone (Mid-year population estimates 2022), with about 1 in 10 having advanced HIV disease (Chihana et al. 2019). Given a SARS-CoV-2 seroprevalence pre-Omicron of 65 per cent in Africa (Lewis et al. 2022) and over 70 per cent in South Africa (Madhi et al. 2022), it is reasonable to assume that the absolute number of SARS-CoV-2 infections which can lead to SARS-CoV-2 evolution is large, even if their proportion of the total number of infections is small. Such long-term infections that can lead to large unobserved evolutionary changes (Neher 2022).

We have previously reported on the evolution of SARS-CoV-2 from ancestral B.1 lineage virus infection in an individual who was immunosuppressed because of advanced HIV disease. Virus evolved in this individual isolated from a nasopharyngeal swab after 190 days of infection evolved known neutralizing antibody escape mutations which reduced neutralization by convalescent sera from people infected with ancestral SARS-CoV-2. However, it became more sensitive to neutralization by sera from people infected with the Beta variant. The evolved virus was also much more resistant to neutralization by Delta variant infection-elicited sera, although there was no evidence that a Delta variant virus infected the immunosuppressed participant (Cele et al. 2022). While this data showed that advanced HIV disease immunosuppression can lead to long-term SARS-CoV-2 infection and evolution of escape from neutralizing antibodies, several aspects were not investigated: viruses from intermediate times in the infection were not tested to determine whether evolution involved a stepwise accumulation of neutralization escape mutations or occurred in other ways. In addition, viral properties such as induction of cell-to-cell fusion, viral replication, and degree of cell death upon infection were not measured between the early and late infection isolates to determine whether these aspects of infection also changed during the evolutionary process.

Here we used intermediate viral isolates from the prolonged SARS-CoV-2 infection to determine how neutralizing antibody escape was acquired. Surprisingly, we found that escape from ancestral and Delta infection elicited antibodies first occurred in a sub-lineage of the infecting virus which was replaced with a second sub-lineage in an apparent evolutionary sweep similar to sweeps seen at the population level with variants of concern. This second sub-lineage was initially more sensitive to neutralization by both ancestral SARS-CoV-2 and Delta infection elicited sera, but then evolved resistance to these sera and increased neutralization sensitivity to Beta-variant infection elicited sera. The first and second sub-lineage did not have spike mutations in common. The virus isolated at day 190 post-diagnosis also evolved higher cell-to-cell fusogenicity, faster replication, and induced more cell death relative to the virus isolated soon after diagnosis at day 6.

These results indicate that neutralizing antibody escape may evolve rapidly and follow multiple trajectories in one immunosuppressed individual. SARS-CoV-2 may also evolve increased fusogenicity and replication, and induce higher cell death, aspects usually associated with higher pathogenicity (Mlcochova et al. 2021; Barut et al. 2022; Hui et al. 2022; Meng et al. 2022; Saito et al. 2022; Suzuki et al. 2022; Willett et al. 2022; Hyams et al. 2023). This may indicate that the decreased pathogenicity likely observed with the Omicron BA.1 variant (Barut et al. 2022; Hui et al. 2022; Madhi et al. 2022; Meng et al. 2022; Sigal 2022; Suzuki et al. 2022; Whitaker et al. 2022; Willett et al. 2022; Wolter et al. 2022; Hyams et al. 2023) may not necessarily evolve in prolonged infection during immunosuppression.

## Results

We isolated live virus from an individual with advanced HIV disease infected with ancestral B.1 lineage SARS-CoV-2 with the D614G substitution who was enrolled in our longitudinal cohort in September 2020, before the discovery of the first variants of concern (Cele et al. 2022). Participants in the study were repeatedly sampled within the first month post-diagnosis, and subsequently at 3-month intervals (Karim et al. 2021a). All participants who were HIV viremic were offered a regimen of tenofovir, lamivudine, and dolutegravir (TLD) as well as adherence counselling. Participants were seen on an outpatient basis for most study visits and the participant presented here had poor adherence to antiretroviral therapy for the period of the prolonged SARS-CoV-2 infection (Cele et al. 2022).

**Figure 1.**
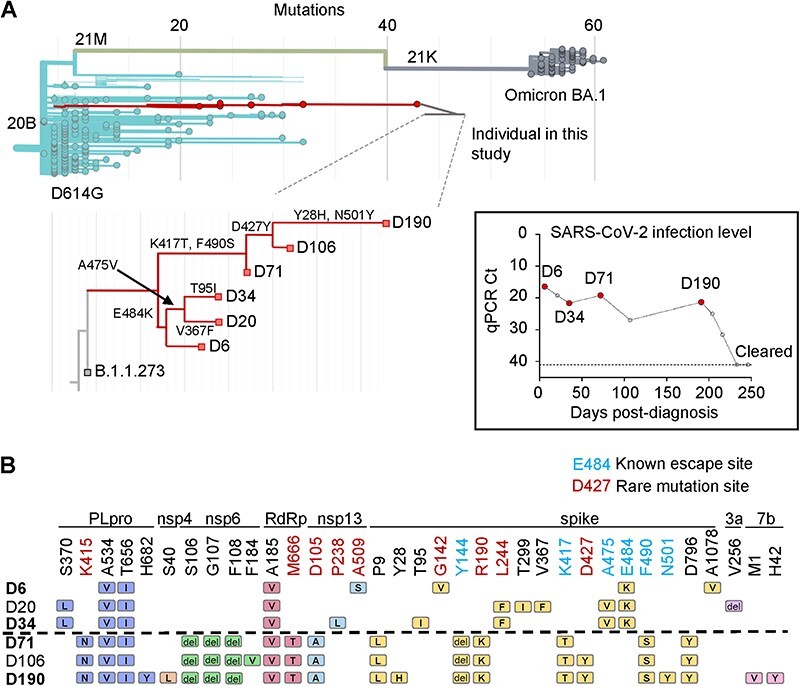
Evolution of SARS-CoV-2 from ancestral virus in an immunosuppressed individual. (A) Phylogenetic tree highlighting isolates from the prolonged infection tested in this study. Magnified section shows relationship between isolates and selected spike mutations which define each bifurcation. Modified from Nextclade. Inset: SARS-CoV-2 qPCR cycle threshold (*Ct*) values over time for the infection. Each point represents a study visit. Horizontal line denotes threshold of detection. (B) Substitutions and deletions in D6, D20, D34, D71, D106, and D190 isolates relative to the B.1.1.273 ancestral SARS-CoV-2 lineage. Rows represent the viral isolates and columns represent mutations in papain-like protease (PLpro), non-structural proteins (nsp) 4, 6, and 13, the RNA dependent RNA polymerase (RdRp), spike, and open reading frames (ORF) 3a and 7b. Amino acids in blue are known sites of neutralizing antibody escape mutations. Amino acids in red are private mutations having a global prevalence below 0.01 percent according to the Stanford University Coronavirus Antiviral and Resistance Database used in the analysis (https://covdb.stanford.edu/).

Continuous high-titre SARS-CoV-2 infection was detected by qPCR from combined nasopharyngeal and oropharyngeal swabs for a period of 6 months (Fig. 1A inset). The first virus isolation was performed on day 6 post-diagnosis, 16 days after self-reported symptom onset (isolate designated D6). Virus was isolated from other study visits including at day 20 (D20), day 34 (D34), day 71 (D71), day 106 (D106), and day 190 (D190). Isolations were performed using an initial passage in H1299-ACE2 cells followed by two passages in unmodified Vero-E6 cells (see ‘Materials and Methods’ section). However, the D6 viral stock showed the R682W mutation in spike, which is usually an *in vitro* derived mutation in the furin cleavage site (Cele et al. 2022). It was re-isolated in this study by two passages in Vero-TMPRSS2 cells (see ‘Materials and Methods’ section), which produced a viral stock without the R682W mutation. All experiments described here are with the isolate that does not have the spike R682W mutation, except for the timelapse microscopy and surface expression results in the H1299-ACE2 cell line presented as Supplementary Data which were performed prior to re-isolation.

Phylogenetic analysis showed a pattern consistent with the presence of a single continuous ancestral virus infection from the B.1.1.273 ancestral SARS-CoV-2 lineage (Fig. 1A). However, higher resolution of the phylogenetic tree of the isolated sequences showed a pattern with two sub-lineages, with one sub-lineage consisting of D6, D20, and D34 and a second sub-lineage consisting of D71, D106, and D190 viruses (Fig. 1A, zoom-in).

Sequences of the viral isolates indicated that within each putative sub-lineage, there was some stepwise accumulation of mutations in SARS-CoV-2 spike and these included mutations known to confer neutralization escape or an advantage in replication (Fig. 1B). The first sub-lineage, which already contained the E484K neutralization escape mutation, evolved A475V in the receptor binding domain of spike known to mediate escape from ancestral SARS-CoV-2 elicited neutralizing antibodies (Greaney, Starr, and Bloom 2022; Cao et al. 2023) (see https://jbloomlab.github.io/SARS2-RBD-escape-calc/). The second sub-lineage, which already contained the neutralization escape mutations K417T and F490S (https://jbloomlab.github.io/SARS2-RBD-escape-calc/) and the N-terminal neutralization escape deletion at 144 (McCallum et al. 2021), evolved spike N501Y, known to increase SARS-CoV-2 infectivity and to a lesser degree neutralization escape (Liu et al. 2022). Strikingly, the two sub-lineages (separated by a horizontal dashed line in Fig. 1B) had no spike mutations in common.

To examine the effects of these mutations on neutralization, we performed live virus neutralization assays using sera from convalescent study participants who were infected with either ancestral SARS-CoV-2 with the D614G spike substitution (D614G), the Beta variant, or the Delta variant (see Supplementary Tables S1–S6 for participant summary characteristics and per participant neutralization values). We tested the early viral isolate D6, the viral isolates from intermediate timepoints D34 and D71 (where D34 was the last detected isolate of the first sub-lineage and D71 was the first detected isolate of the second sub-lineage), as well as D190, the last isolate of the second sub-lineage. We measured neutralization in a focus forming assay by quantifying the reduction in the number of foci with sera relative to the no-serum control and calculated the focus reduction neutralization test (FRNT_50_) value, the inverse of the plasma dilution required for 50 per cent reduction in infection focus number.

**Figure 2.**
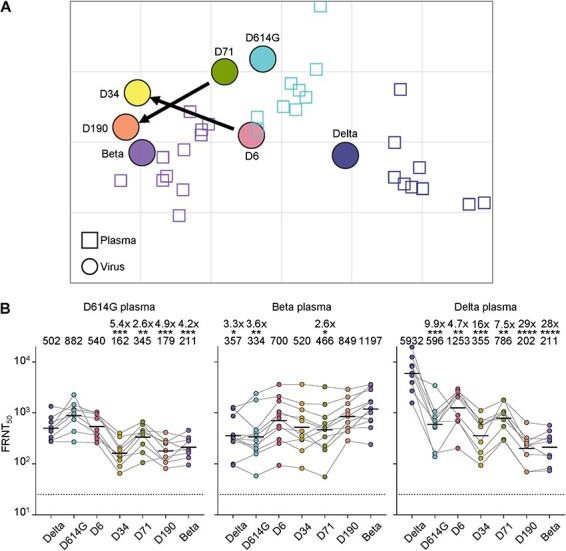
Evolution of escape from neutralizing antibodies. (A) Antigenic cartography using the Racmacs package. Input was neutralization of D614G, Beta, Delta, and the D6, D34, D71, and D190 isolated viruses from the long-term infection, with neutralizing sera from participants infected with ancestral virus (*n* = 9), Beta (*n* = 11), and Delta (*n* = 9) variants. Grid shows antigenic units (AU), where each antigenic unit represents a 2-fold change in the plasma concentration required for 50 per cent neutralization. Squares represent sera from individual participants, and circles represent viruses. Arrow represents shift in antigenicity from D6 to D34 and D71 to D190. (B) Neutralization of the ancestral/D614G, Beta, Delta, and the D6, D34, D71, and D190 isolates with neutralizing immunity elicited by infection with ancestral D614G (left), Beta (middle), and Delta (right) variants. Sera are the same as in panel A. *Y*-axis is neutralization as FRNT_50_ and numbers above each column are geometric mean titer (GMT) FRNT_50_ for the group and the fold-drop relative to FRNT_50_ of homologous virus (D614G for first, Beta for second, and Delta virus for third panel). Significant *P*-values relative to homologous virus were for the D614G sera panel: ***D34 *P* = 0.0002, **D71 *P* = 0.006, ***D190 *P* = 0.0002, ***Beta *P* = 0.0008. For the Beta sera panel: *Delta *P* = 0.01, **D614G *P* = 0.007, *D71 *P* = 0.02. For the Delta sera panel: ***D614G *P* = 0.0002, **D6 *P*= 0.001, ***D34 P = 0.0005, **D71 *P*= 0.002, ****D190 and Beta *P* < 0.0001. All *P*-values by the Wilcoxon Rank Sum test for the comparison to the homologous virus. Data from three independent experiments.

To analyze the results, we performed antigenic cartography using the Racmacs package https://acorg.github.io/Racmacs/articles/intro-to-antigenic-cartography.html) to quantitatively map relationships between D614G, Beta, and Delta sera and the D614G, Beta, Delta, D6, D34, D71, and D190 viruses (Fig. 2A). This method shows the antigenic relationships between viruses and sera in 2 dimensional space, where each square of distance (antigenic unit, AU) is a 2-fold increase in the plasma concentration required for 50 per cent neutralization (Smith et al. 2004). Despite using sera from different participants relative to our previous work (Cele et al. 2022), we obtained very similar patterns, whereby Beta and Delta variants were serologically far apart, while D614G was intermediate between Beta and Delta (Fig. 2A). The D6 isolate was serologically similar to D614G. However, the antigenic distance increased with the D34 isolate, the last isolate of the first sub-lineage. After the sweep of the first sub-lineage, the first isolate of the second sub-lineage, D71, was closer to D614G and Delta than D34. This sub-lineage also evolved increased neutralization escape, and the D190 virus was antigenically far from D614G and Delta and close to Beta virus in its neutralization pattern.

Given that the antigenic cartography combines distances from the three different panels of sera, we also investigated neutralization of each virus by the individual convalescent sera panels (Fig. 2B). We observed that D34 and D190, with FRNT_50_ of 162 (D34) and 179 (D190), show significantly lower neutralization compared to D614G virus (FRNT_50_ = 882) when neutralized by D614G infection elicited neutralizing sera, while D6 (FRNT_50_ = 540) and Delta (FRNT_50_ =502) viruses do not show significantly lower neutralization. D71, despite being close to D614G in the antigenic cartography, does show significant neutralization escape (FRNT_50_ = 345) from D614G infection elicited neutralization, although escape is less than that observed for D34 and D190 viruses. Conversely, D34 (FRNT_50_ = 520) and D190 (FRNT_50_ = 849) do not show significant escape from neutralizing immunity elicited by Beta-variant infection (FRNT_50_ = 1197 for Beta virus), while D614G, Delta, and D71 do show significantly lower neutralization relative to the Beta virus (Delta virus FRNT_50_ = 357, D614G virus FRNT_50_ = 334, and D71 FRNT_50_ = 466). Interestingly, D6 is relatively well neutralized by Beta infection elicited neutralizing immunity (FRNT_50_ = 700). All viruses show significant escape of Delta infection elicited neutralizing immunity relative to Delta virus. D190 and Beta show the strongest escape (FRNT_50_ of 202 and 211 translating to 29- and 28-fold drops, respectively), while D6 and D71 show the lowest escape (FRNT_50_ of 1253 and 786, respectively).

We next investigated how additional viral aspects evolved in the prolonged infection. We examined cell-to-cell fusion after infection, viral replication, and cell death in infected cells. To quantify cell fusion, we used Vero-TMPRSS2 cells which are engineered to express ACE2 and TMPRSS2, allowing for efficient infection. We found that this system led to fusions being present early post-infection by cell-free virus (see Supplementary Movie S1 for timelapse microscopy of uninfected Vero-TMPRSS2 cells and Movie S2 for D614G infected cells) and we could quantify fusions at 10 h post-infection, therefore reducing the effects of replication differences between strains.

**Figure 3.**
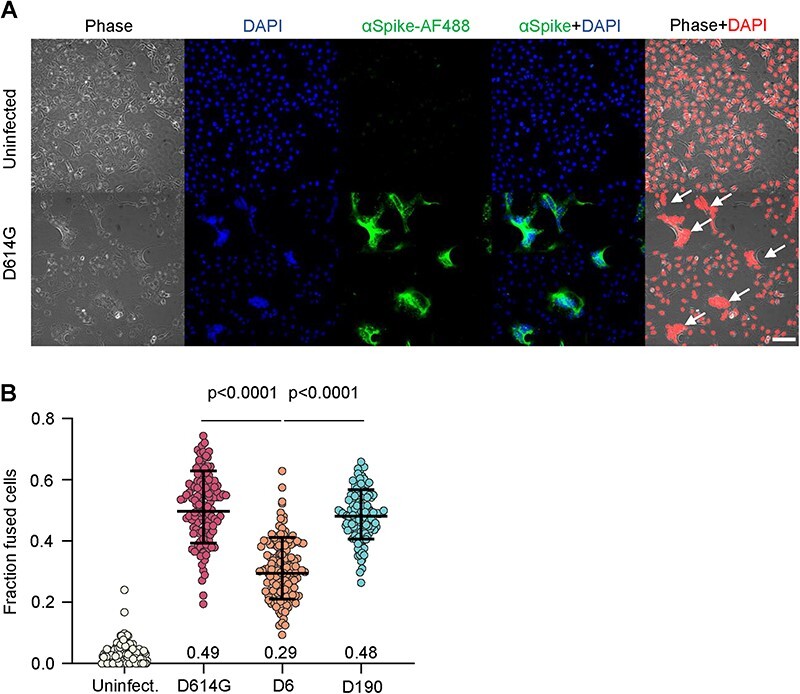
Infection induced cell–cell fusion by immunosuppression-derived viruses versus D614G. (A) Representative fields of view from uninfected (top row) and D614G infected (bottom row) Vero-TMPRSS2 cells. Images were transmitted light (first column), cell nuclei by DAPI (second column), anti-spike AlexaFluor488 conjugated antibody to detect infected cells (third column), overlay of infection and DAPI signal (forth column), and overlay of DAPI (in red for visibility) and transmitted light (fifth column). Arrows indicate fusions. Scale bar is 100 μm. (B) Automatically quantified fraction of fused cells. Each point in the plot is the fraction of fused cells in one field of view from uninfected (left), D614G infected (second from left), D6 infected (second from right), and D190 infected cells (right). *n* = 128 Fields of view were analyzed for each infection condition. Bars are geometric means and standard deviations and numbers are fractions of fused cells per group. Fraction of fused cells was calculated by dividing the nuclear signal from cells scored as fused by total nuclear area per field of view. Data from two independent experiments. All comparisons *P* < 0.0001 except D614G versus D190 (non-significant) by Kruskal–Wallis test with Dunn multiple hypothesis correction.

We confirmed infection using an anti-spike antibody (Fig. 3A). We used distances between DAPI-stained nuclei as our method to quantify fusions. We noted that cell nuclei become clustered together to form a contiguous region of fluorescence during cell fusion (Fig. 3A) and used an automated image analysis pipeline (Supplementary Fig. S1) to detect fused cells and cell number. In the absence of fusion, individual cell nuclei are distinct even in confluent cell culture because they are separated by cellular cytoplasm. We found that the fraction of fusions in the absence of infection was low. Cells infected with D6 (no R682W) had about 29 per cent of cell nuclei in cell fusions at 10 h post-infection. This was a significantly lower fraction of fusions compared to D614G infection, which had 49 per cent of cells in fusions. The fraction of cell–cell fusions detected in D190 infection was 48 per cent, not significantly different from D614G (Fig. 3B). Surprisingly, we did not observe that the R682W mutation reduced fusions (Supplementary Fig. S2).

We also present earlier experiments where we infected human lung epithelial H1299-ACE2 cells with D6 with the R682W mutation (Supplementary Fig. S3A). These are the only experiments presented here which use this earlier D6-R682W isolate. We detected fusions by time-lapse microscopy of cells with YFP labelled nuclei (see Supplementary Movie S3 for timelapse of uninfected H1299 cells and Movie S4 for timelapse of cells infected with D614G) and compared D6-R682W with D190, D614G, and Omicron BA.1. Like Vero-TMPRSS2 cells, uninfected cell culture had a low frequency of fusions which did not increase over time. Infection by the D614G virus showed an increasing frequency of fusions, with about 40 per cent of cell nuclei belonging to fused cells by 36 h post-infection (Supplementary Fig. S3B). The frequency of fusions was lower with BA.1 infection throughout and reached less than half of that seen in the D614G virus infection. The pattern in D6 virus infections was similar to BA.1. D190 showed a fusion frequency intermediate between BA.1 and D614G (Supplementary Fig. S3B). Cell fusion occurs through an interaction between SARS-CoV-2 spike on the infected cell surface and ACE2 on the adjacent cell (Buchrieser et al. 2020; Rajah et al. 2021, 2022). We observed that spike cell surface expression increased in D190 compared to D6 infected cells (Supplementary Fig. S4), possibly accounting for some of the higher fusogenicity of D190 relative to D6.

**Figure 4.**
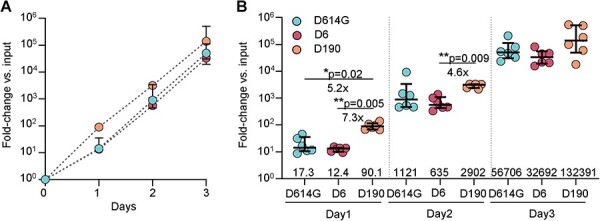
Replication in H1299-ACE2 cells. (A) Fold-change in SARS-CoV-2 viral copies was determined using qPCR cycle threshold over 3 days of infection in H1299-ACE2 infected by 10 focus forming units per 10^6^ cells using either ancestral SARS-CoV-2, D6, or D190. Shown are median and interquartile ranges for six measurements from two independent experiments, with fold-change calculated as 2^((mean(^*^Ct^*  ^input)—^*^Ct^*  ^sample)^, with the input being the most dilute sample. (B) Fold change relative to D190 and significance of replication differences for data from panel A. Shown are median and interquartile ranges and the six measurements for each timepoint per viral strain. *P*-values determined by the Kruskal–Wallis test with Dunn multiple hypothesis correction per day relative to D190.

To determine whether differences in viral replication were evolved in the prolonged infection, we measured replication of D614G, D6, and D190 in H1299-ACE2 cells, where H1299 cells are reported to have an interferon response (Machitani et al. 2017; Reisländer et al. 2019; Gao et al. 2021; Zhang et al. 2021; Duncan et al. 2023). We observed that D190 initially replicated significantly faster than either D6 or D614G (Fig. 4A). However, the significance of the differences was reduced on day 2 post-infection and was absent on day 3 post-infection (Fig. 4B).

**Figure 5.**
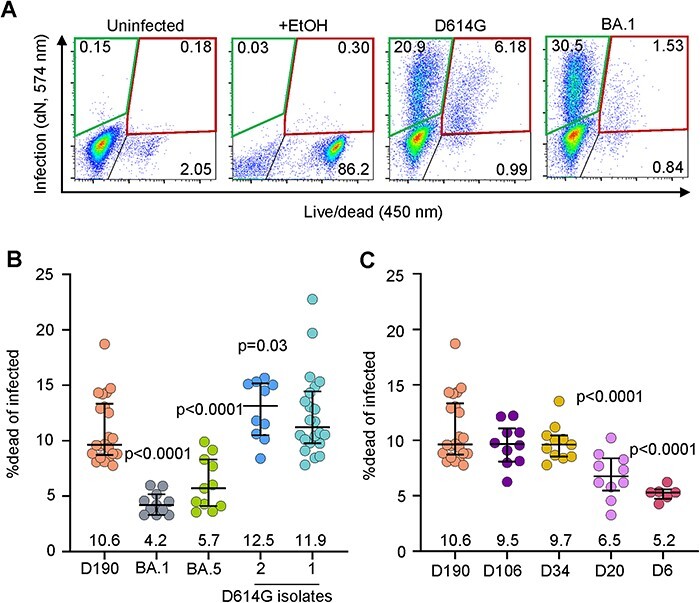
SARS-CoV-2 induced cell death. (A) Flow cytometry gating strategy. First panel shows uninfected cells, second panel shows 80 per cent ethanol-treated cells (positive control), and third and fourth panels show live infected cells (green gate, top left) and dead infected cells (red gate, top right) 24 h post-infection with D614G and Omicron BA.1. Numbers represent percentages of cells in the corresponding quadrants. (B) Fraction of dead cells 24 h post-infection in infections by D190, Omicron subvariants BA.1 and BA.5, and two independent isolates of ancestral/D614G virus. (C) Fraction of dead cells 24 h post-infection in infections by D190, D106, D34, D20, and D6. Horizontal bars represent medians with interquartile ranges of 10–22 replicates from 3–8 independent experiments with all experiments containing D190 and the D614G (isolate 1) for reference. *P*-values were determined by the Kruskal–Wallis test with Dunn multiple comparisons correction, with all comparisons to D190.

We also measured infection-induced cell death at 24 h post-infection in H1299-ACE2 cells. We used this relatively early timepoint to avoid saturating infection (Sigal et al. 2011; Jackson et al. 2018), which happens later (see Supplementary Movie S4). Infection was detected by staining for SARS-CoV-2 nucleocapsid and death by co-staining with a death detection dye (Fig. 5A). We tested the B.1 isolate used in the previous experiments described here (B.1 lineage) and in addition a second isolate of ancestral SARS-CoV-2 with the D614G substitution (B.1.1.117, see ‘Materials and Methods’ section). We also tested the Omicron subvariants BA.1 and BA.5, and the D6, D20, D34, D106, and D190 isolates. D614G isolates caused similar cell death (12.5 per cent and 11.9 per cent, Fig. 5B). Infected cell death was 5.6 per cent for D6 virus infection, 6.8 per cent for D20, 4.2 per cent for Omicron BA.1, and 5.7 per cent for Omicron BA.5 (Fig. 5B**–**C). D190 infection resulted in 10.6 per cent dead infected cells, significantly higher than D6, D20, BA.1 and BA.5 and similar to the ancestral virus strains and the D106 viral isolate from the second sub-lineage, as well as the last isolate (D34) from the first sub-lineage (9.6 per cent and 9.7 per cent, respectively, Fig. 5B**–**C).

## Discussion

Here we described a prolonged SARS-CoV-2 infection in an immunosuppressed individual because of advanced HIV disease. The virus, which originated from an ancestral SARS-CoV-2 infection, showed evolution of neutralization escape from immunity induced by the virus originating the infection. In addition, the evolved virus showed higher fusogenicity, faster initial replication, and resulted in more cell death. The evolution involved some stepwise accumulation of mutations, but most changes happened by an abrupt shift from one sub-lineage to another that resembled the evolutionary sweeps seen with variants. Such sub-lineages were observed in a previous report (Chaguza et al. 2023) so may not be unique. The earliest isolates from each sub-lineage showed a neutralization profile similar to ancestral virus despite containing some known neutralizing antibody escape mutations such as E484K (first lineage) and K417T and F490S (second lineage). The presence of multiple mutations in the sample isolated on day 6 post-diagnosis, 16 days post-self-reported symptom onset, may argue that the infection happened some time before the self-reported symptom onset date.

Both the first and second sub-lineages were classified as B.1.1.273 by Nextclade and the day 34 and later samples were collected through the Beta-variant infection wave (Cele et al. 2022). Therefore, re-infection with ancestral virus is an unlikely explanation for the second sub-lineage, as it would have had to be an infection with a very similar virus several months after the first infection, when very few infections were by ancestral SARS-CoV-2. Possibly, it could be a re-infection from an immunosuppressed household contact, although we have currently no evidence for this.

The higher sensitivity to neutralizing antibodies elicited by ancestral SARS-CoV-2 and the Delta variant by the first virus sampled in the second sub-lineage (D71) is surprising, but the sweep may be explained by other selective advantages over the first sub-lineage, including the 106–108 deletion in nsp 6 which also arose independently in the Alpha, Beta, and Gamma variants and the Omicron BA.2 subvariant, but which is absent in ancestral D614G SARS-CoV-2 (https://covdb.stanford.edu/variants/omicron_ba_2/). This deletion results in evasion of the interferon type 1 response and may therefore enhance replication (Xia et al. 2020). The spike N501Y substitution present in the D190 isolate may also increase fitness by enhancing spike affinity to the ACE2 receptor (Liu et al. 2022). It would have been difficult to predict the increase in neutralization escape between the D71 and D190 isolate based on the added spike mutations. These include N501Y and D427Y in the receptor-binding domain, and Y28H close to the N-terminal domain supersite (McCallum et al. 2021). Neither N501Y nor D427Y are reported to lead to strong escape from ancestral SARS-CoV-2 elicited neutralization (https://jbloomlab.github.io/SARS2-RBD-escape-calc/), while Y28H is shown to lead to some escape from monoclonal antibodies (McCallum et al. 2021).

Higher cell–cell fusogenicity in D190 may give the virus a selective advantage as it may facilitate cellular transmission and immune evasion. It is enhanced in Alpha, Beta, and Delta variants relative to ancestral virus (Buchrieser et al. 2020; Rajah et al. 2021, 2022). The genetic changes responsible are difficult to pinpoint, given there are 12 differences in the spike protein alone between D190 and the less fusogenic D6 isolate. Disease severity in the Omicron subvariant period has been reduced relative to pre-Omicron, and this may be both due to increased immunity in the population, as well as reduced pathogenicity of the virus (Sigal 2022). Higher viral replication, fusogenicity, and cell death may lead to increased pathogenicity. We note that D190 is not more fusogenic and does not cause more cell death than the ancestral D614G virus. Therefore, while cell fusion and cell death were increasing relative to the virus isolated early in infection, they were still similar or lower than for ancestral SARS-CoV-2 for the last isolated virus.

A limitation of the study is that our results are from one case. However, the first and second sub-lineage from this infection seemed to have followed independent evolutionary trajectories and the degree of D34 (late first sub-lineage virus) and D190 (late second sub-lineage virus) escape from ancestral SARS-CoV-2 and Delta elicited neutralizing immunity is similar to each other and the Beta-variant virus, possibly indicating convergent evolution of escape from neutralizing antibodies. The recent emergence of the Omicron BA.2.86 subvariant (Hu et al. 2023; Lasrado et al. 2023; Lassaunière et al. 2023; Qu et al. 2023; Sheward et al. 2023; Uriu et al. 2023; Wang et al. 2023; Yang et al. 2023), in the absence of detected intermediates, from an infection that may have lasted over a year and which likely occurred in Southern Africa (Khan et al. 2023), may indicate that evolution of SARS-CoV-2 in prolonged infection is still an ongoing process.

## Materials and methods

### Informed consent and ethical statement

Swabs for the isolation of ancestral (D614G.1 and D614G.2), Beta, Delta, D6, D20, D34, D71, D106, and D190 viruses, and all blood samples from participants infected with D614G, Beta, and Delta variants used to test virus neutralization were obtained after written informed consent from adults with PCR-confirmed SARS-CoV-2 infection who were enrolled in a prospective cohort study at the Africa Health Research Institute approved by the Biomedical Research Ethics Committee at the University of KwaZulu–Natal (reference BREC/00001275/2020). The Omicron/BA.1 was isolated from a residual swab sample with SARS-CoV-2 isolation from the sample approved by the University of the Witwatersrand Human Research Ethics Committee (HREC) (ref. M210752). The sample to isolate Omicron/BA.5 was collected after written informed consent as part of the COVID-19 transmission and natural history in KwaZulu-Natal, South Africa: Epidemiological Investigation to Guide Prevention and Clinical Care in the Centre for the AIDS Programme of Research in South Africa (CAPRISA) study and approved by the Biomedical Research Ethics Committee at the University of KwaZulu–Natal (reference BREC/00001195/2020, BREC/00003106/2021).

### Reagent availability statement

Isolates and raw image files are available upon reasonable request. Sequences of isolated SARS-CoV-2 used in this study have been deposited in GISAID with accession as described in Supplementary Table S7. The Matlab 2019b-based image analysis script used for quantifying fusions is included in the submission.

### Whole-genome sequencing and phylogenetic and sequence analysis

For all samples except re-isolated D6 without the spike R682W mutation, RNA was extracted on an automated Chemagic 360 instrument, using the CMG-1049 kit (Perkin Elmer, Hamburg, Germany). The RNA was stored at −80˚C prior to use. Libraries for whole-genome sequencing were prepared using either the Oxford Nanopore Midnight protocol with Rapid Barcoding or the Illumina COVIDseq Assay. For the Illumina COVIDseq assay, the libraries were prepared according to the manufacturer’s protocol. Briefly, amplicons were tagmented, followed by indexing using the Nextera UD Indexes Set A. Sequencing libraries were pooled, normalized to 4 nM, and denatured with 0.2 N sodium acetate. An 8 p.m. sample library was spiked with 1 per cent PhiX (PhiX Control v3 adaptor-ligated library used as a control). We sequenced libraries on a 500-cycle v2 MiSeq Reagent Kit on the Illumina MiSeq instrument (Illumina). On the Illumina NextSeq 550 instrument, sequencing was performed using the Illumina COVIDSeq protocol (Illumina Inc, USA), an amplicon-based next-generation sequencing approach. The first-strand synthesis was carried using random hexamers primers from Illumina and the synthesized cDNA underwent two separate multiplex PCR reactions. The pooled PCR amplified products were processed for tagmentation and adapter ligation using IDT for Illumina Nextera UD Indexes. Further enrichment and cleanup was performed as per protocols provided by the manufacturer (Illumina Inc). Pooled samples were quantified using Qubit 3.0 or 4.0 fluorometer (Invitrogen Inc.) using the Qubit dsDNA High Sensitivity assay according to manufacturer’s instructions. The fragment sizes were analyzed using TapeStation 4200 (Invitrogen). The pooled libraries were further normalized to 4 nM concentration and 25 μL of each normalized pool containing unique index adapter sets were combined in a new tube. The final library pool was denatured and neutralized with 0.2 N sodium hydroxide and 200 mM Tris–HCL (pH7), respectively. A 1.5 p.m. sample library was spiked with 2 per cent PhiX. Libraries were loaded onto a 300-cycle NextSeq 500/550 HighOutput Kit v2 and run on the Illumina NextSeq 550 instrument (Illumina, San Diego, CA, USA). For Oxford Nanopore sequencing, the Midnight primer kit was used as described by Freed and Silander55. cDNA synthesis was performed on the extracted RNA using LunaScript RT mastermix (New England BioLabs) followed by gene-specific multiplex PCR using the Midnight Primer pools which produce 1200 bp amplicons which overlap to cover the 30-kb SARS-CoV-2 genome. Amplicons from each pool were pooled and used neat for barcoding with the Oxford Nanopore Rapid Barcoding kit as per the manufacturer’s protocol. Barcoded samples were pooled and bead-purified. After the bead clean-up, the library was loaded on a prepared R9.4.1 flow-cell. A GridION X5 or MinION sequencing run was initiated using MinKNOW software with the base-call setting switched off. We assembled paired-end and nanopore.fastq reads using Genome Detective 1.132 (https://www.genomedetective.com) which was updated for the accurate assembly and variant calling of tiled primer amplicon Illumina or Oxford Nanopore reads, and the Coronavirus Typing Tool. For Illumina assembly, GATK HaploTypeCaller—min-pruning 0 argument was added to increase mutation calling sensitivity near sequencing gaps. For Nanopore, low-coverage regions with poor alignment quality (<85 per cent variant homogeneity) near sequencing/amplicon ends were masked to be robust against primer drop-out experienced in the Spike gene, and the sensitivity for detecting short inserts using a region-local global alignment of reads, was increased. In addition, we also used the wf_artic (ARTIC SARS-CoV-2) pipeline as built using the nextflow workflow framework. In some instances, mutations were confirmed visually with .bam files using Geneious software V2020.1.2 (Biomatters). The reference genome used throughout the assembly process was NC_045512.2 (numbering equivalent to MN908947.3). For lineage classification, we used the widespread dynamic lineage classification method from the ‘Phylogenetic Assignment of Named Global Outbreak Lineages’ (PANGOLIN) software suite (https://github.com/hCoV-2019/pangolin).

For the D6 outgrowth sample in TMPRSS2 cells, Oxford Nanopore sequencing was performed. RNA was manually extracted from 140 µl using the QIAamp Viral RNA Kit (Qiagen) as per the manufacturer’s protocols. All RNA extractions were measured using Qubit fluorimeter kits (Thermo Scientific). The cDNA synthesis was performed using LunaScript RT mastermix (New England BioLabs) followed by whole-genome multiplex PCR using the Midnight Primer pools v3 (EXP-MRT001, Oxford Nanopore) that produce 1200-bp amplicons. The amplified products for each pool were combined and library prepared using Oxford Nanopore Rapid Barcoding kit (SQK-RBK110.96, Oxford Nanopore). The barcoded samples were pooled and cleaned using magnetic beads and loaded on an R9.4.1 flow cell for 8 h sequencing on a MinION device. The raw data were processed using Guppy basecaller and Guppy barcoder (Oxford Nanopore) for basecalling and demultiplexing. The final consensus sequences were obtained using the Epi2Me desktop agent ARTIC pipeline (Oxford Nanopore). The lineage assignment was determined using Nextclade. In this case where a mutation site had an indeterminate sequence, we called the mutation if it was present in the earlier D6 isolate with the R682W mutation.

To determine substitutions and deletions in SARS-CoV-2 proteins, sequence was input into the sequence analysis application in the Stanford Coronavirus Antiviral and Resistance Database (https://covdb.stanford.edu/sierra/sars2/by-sequences/).

### Phylogenetic analysis

The consensus sequences were loaded into the pre-release of Nextclade web v3 and analyzed using the dataset ‘nextstrain/sars-cov-2/wuhan-hu-1’ with release tag 2023–10-26T12:00:00Z. This version constructs a parsimony based phylogenetic tree of the query sequences embedded in the reference tree containing most common pango lineages.

### Cells

The H1299-E3 (H1299-ACE2, clone E3, H1299 originally from ATCC as CRL-5803) cell line was derived from H1299 as described in our previous work (Cele et al. 2021a, 2021b). The H1299-E3 cells were propagated in growth medium consisting of complete Roswell Park Memorial Institute (RPMI) 1640 medium with 10 per cent fetal bovine serum (Hyclone) containing 10 mM of hydroxyethylpiperazine ethanesulfonic acid (HEPES), 1 mM sodium pyruvate, 2 mM l-glutamine and 0.1 mM nonessential amino acids (all Sigma-Aldrich). Cells were passaged every second day. For virus isolation, Vero E6 cells (originally ATCC CRL-1586, obtained from Cellonex in South Africa) were propagated in complete growth medium consisting of Dulbecco’s Modified Eagle Medium (DMEM) with 10 per cent fetal bovine serum (Hyclone) containing 10 mM of HEPES, 1 mM sodium pyruvate, 2 mM l-glutamine and 0.1 mM nonessential amino acids (all Sigma-Aldrich). Vero E6 cells were passaged every 3–4 days. The VeroE6 cells expressing TMPRSS2 and ACE2 (VeroE6-TMPRSS2), originally BEI Resources, NR-54,970 were used for virus re-expansion of the virus from the day 6 swab and fusion assay. The Vero-TMPRSS2 cell line was propagated in the same way as VeroE6 cells.

### Virus isolation

All work with live virus was performed in Biosafety Level 3 containment using protocols for SARS-CoV-2 approved by the Africa Health Research Institute Biosafety Committee. ACE2-expressing H1299-E3 cells were seeded at 4.5 × 10^5^ cells in a 6-well plate well and incubated for 18–20 h. After one Dulbecco’s phosphate-buffered saline (DPBS) wash, the sub-confluent cell monolayer was inoculated with 500 μl universal transport medium from swabs diluted 1:1 with growth medium filtered through a 0.45 μm filter. Cells were incubated for 1 h. Wells were then filled with 3 ml complete growth medium. After 4 days of infection (completion of passage 1 (P1)), cells were trypsinized (Sigma-Aldrich), centrifuged at 300 rcf for 3 min and resuspended in 4 ml growth medium. Then all infected cells were added to Vero E6 cells that had been seeded at 1.5 × 10^5^ cells/ml, 20 ml total, 18–20 h earlier in a T75 flask for cell-to-cell infection. The co-culture of ACE2-expressing H1299-E3 and Vero E6 cells was incubated for 1 h and the flask was filled with 20 ml of complete growth medium and incubated for 4 days. The viral supernatant from this culture (passage 2 (P2) stock) was used for experiments. For re-isolation of D6 virus without the R682W mutation, VeroE6-TMPRSS2 cells were seeded at 4.5 × 10^5^ cells in a 6-well plate well and incubated for 18–20 h pre-infection. After one DPBS wash, the sub-confluent cell monolayer was inoculated with 150 μl with universal transport medium which contained the swab, diluted with 600 μl growth medium filtered through a 0.45 μm filter. Cells were incubated for 2 h. Wells were then filled with 3 ml complete growth medium. After 2 days of infection (completion of P1), supernatant was collected, cells were trypsinized, centrifuged at 300 rcf for 3 min, and resuspended in 3 ml growth medium. Infected cells and supernatant were added to VeroE6-TMPRSS2 cells that had been seeded at 1.5 × 10^5^ cells/ml, 20 ml total, 18–20 h earlier in a T75 flask for cell-to-cell infection. The co-culture was incubated for 1 h and the flask was filled with 20 ml of complete growth medium and incubated for 1 day. The viral supernatant from this culture (P2 stock) was used for experiments which tested D6 without the R682W spike mutation.

### Live virus neutralization assay

H1299-E3 cells were plated in a 96-well plate (Corning) at 30,000 cells per well 1 day pre-infection. Plasma was separated from EDTA-anticoagulated blood by centrifugation at 500 rcf for 10 min and stored at −80°C. Aliquots of plasma samples were heat-inactivated at 56°C for 30 min and clarified by centrifugation at 10,000 rcf for 5 min. Virus stocks were used at ∼50–100 focus-forming units per microwell and added to diluted plasma. Antibody–virus mixtures were incubated for 1 h at 37°C, 5 per cent CO_2_. Cells were infected with 100 μl of the virus–antibody mixtures for 1 h, then 100 μl of a 1× RPMI 1640 (Sigma-Aldrich, R6504), 1.5 per cent carboxymethylcellulose (Sigma-Aldrich, C4888) overlay was added without removing the inoculum. Cells were fixed 18 h post-infection using 4 per cent PFA (Sigma-Aldrich) for 20 min. Foci were stained with a rabbit anti-spike monoclonal antibody (BS-R2B12, GenScript A02058) at 0.5 μg/ml in a permeabilization buffer containing 0.1 per cent saponin (Sigma-Aldrich), 0.1 per cent BSA (Sigma-Aldrich) and 0.05 per cent Tween-20 (Sigma-Aldrich) in PBS. Plates were incubated with primary antibody overnight at 4 °C, then washed with wash buffer containing 0.05 per cent Tween-20 in PBS. Secondary goat anti-rabbit HRP-conjugated antibody (Abcam ab205718) was added at 1 μg/ml and incubated for 2 h at room temperature with shaking. TrueBlue peroxidase substrate (SeraCare 5510–0030) was then added at 50 μl per well and incubated for 20 min at room temperature. Plates were imaged in an ImmunoSpot Ultra-V S6-02-6140 Analyzer ELISPOT instrument with BioSpot Professional built-in image analysis (C.T.L).

### Statistics and fitting

Fitting was performed using MATLAB v.2019b. Neutralization data were fit to:


(1) $$Tx = 1/\left( {1 + \left( {D/ID_{50}} \right)} \right)$$

Here Tx is the number of foci normalized to the number of foci in the absence of plasma on the same plate at dilution D and ID_50_ is the plasma dilution giving 50 per cent neutralization. FRNT_50_ = 1/ID_50_. Values of FRNT_50_ <1 are set to 1 (undiluted), the lowest measurable value. We note that the most concentrated plasma dilution was 1:25 and therefore FRNT_50_ < 25 were extrapolated.

The 95 per cent confidence intervals on the median in the time-lapse microscopy data were calculated by first ranking the values in ascending order, then finding the ranks of the lower and upper confidence interval by:


(2) $${\rm LR}{\ } = {\ }n/2 - 1.96{}^{\displaystyle\surd} \left( {n/4} \right)$$

(3) $${\rm UR}{\ } = {\ }n/2 + 1.96{}^{\displaystyle\surd} \left( {n/4} \right)$$

Here LR is lower 95 per cent interval index in the ranked vector, UR is the upper 95 per cent interval index in the ranked vector. Values at indexes LR and UR were the lower 95 per cent and upper 95 per cent confidence intervals, respectively. Other statistical tests, measures of central tendency and confidence intervals were performed in GraphPad Prism version 9.4.1.

### Fusion assay in Vero-TMPRSS2 cells

The 6-well glass bottom plates (MatTek) were coated with 300 µl of 0.001 per cent fibronectin (Sigma-Aldrich) in DPBS^−/−^ (Gibco), incubated for 90 min, then washed 3× with DPBS. Vero-TMPRSS2 cells were then immediately plated at 80,000 cells per coated well. One day post-plating the cells were infected at 4000 focus-forming units in 1 ml growth media per well. Cell–virus mixtures were incubated for 1 h at 37°C, 5 per cent CO_2_ then an additional 1 ml of growth media was added. Cells were incubated for 10 h at 37°C, 5 per cent CO_2_. All media was then removed and cells were fixed with 2 ml 4 per cent PFA for 20 min at room temperature. PFA was removed and cells washed 3× in 2 ml PBST (PBS + 0.5 per cent Tween 20). Anti-SARS-CoV-2 spike protein RBD Alexa Fluor 488 fluorescently conjugated antibody (Abcam) was diluted 1:250 with perm/wash buffer [0.1 per cent saponin (Sigma-Aldrich), 0.1 per cent BSA (Sigma-Aldrich), and 0.05 per cent Tween-20 (Sigma-Aldrich) in PBS] and 300 µl added over the coverslip area and incubated for 30 min in the dark at room temperature. Wells were then washed 3× with 2 ml PBST. 4′,6-diamidino-2-phenylindole, (DAPI) (Akoya Biosciences) was diluted at 1 drop in 3 ml DPBS and 300 µL added over the coverslip area and incubated 5 min in the dark at room temperature. Wells were then washed 3× with 2 ml DPBS and cells imaged in 2 ml DPBS to prevent drying. Cells were imaged using a Metamorph-controlled Nikon TiE motorized microscope (Nikon Corporation) using a 20×, 0.75 NA phase objective, 365 nm (for DAPI) and 490 nm (Alexa Fluor 488) LED excitation wavelengths from the pE-4000 Universal Fluorescence Illumination System (CoolLED) and a quad cube for color bands DAPI, fluorescein isothiocyanate, Tetramethylrhodamine, and Cy5 fluorescence (Nikon). Images were captured using an 888 EMCCD camera (Andor). For each well, 40 randomly chosen fields of view were captured per infection.

### Time-lapse microscopy

The 6-well glass bottom plates (MatTek) were coated with 300 µl of 0.001 per cent fibronectin (Sigma-Aldrich) in DPBS^−/−^ (Gibco), incubated for 90 min, then washed 3× with DPBS. H1299-E3 cells were then immediately plated at 60,000 cells per coated well. The next day the cells were infected at 1000 focus-forming units in 1 ml growth media per well. Cell–virus mixtures were incubated for 1 h at 37°C, 5 per cent CO_2_ then an additional 1 ml of growth media was added. Infections were imaged using a Metamorph-controlled Nikon TiE motorized microscope (Nikon Corporation) in a Biosafety Level 3 Facility with a 20×, 0.75 NA phase objective. Images were captured using an 888 EMCCD camera (Andor). Temperature (37°C), humidity, and CO_2_ (5 per cent) were controlled using an environmental chamber (OKO Labs). Excitation source was 488 laser line and emission was detected through a Semrock Brightline quad band 440–40 /521–21/607–34/700–45 nm filter. For each well, 12 randomly chosen fields of view were captured every 10 min.

### Image analysis

Timelapse microscopy images were analysed using custom MATLAB v.2019b (MathWorks) script and using the MATLAB Image Analysis Toolbox. For each frame in the movie, both the transmitted light and fluorescent images were used. A coarse segmentation was first performed using the transmitted light image of the cells. Images were flatfield corrected and contrast was enhanced by setting the top and bottom 1 per cent of all pixel intensities to 1 and 0, respectively. The built-in function ‘rangefilt’ was used to determine areas of high contrast (where high-pixel intensities were immediately adjacent to low pixel intensities) in the image which corresponded to cell borders. Processed images were also median filtered and holes to filled within segmented objects. The image was then thresholded to remove background signal and obtain a mask of areas occupied by cells. The mask generated from the transmitted light image was then used to remove objects that were not in areas occupied by cells in the fluorescence images corresponding to the transmitted light images. Fluorescence images were then processed using flatfield correction, contrast enhancement, and median filtering. Fluorescent cell nuclei in the YFP channel were used to generate a binary mask for contiguous objects in each image after thresholding. Each object was categorized as multi-nucleate or uni-nucleate based on pixel area, where the threshold for a single nucleus was calculated as the mean area of objects/nuclei in the uninfected condition, at 12 h post-movie start, + 3 standard deviations of the mean. The fraction of cells in fusions was calculated by dividing the total pixel area of objects above the single-nucleus threshold by the total pixel area occupied by nuclei in the same frame. The number of nuclei was calculated by dividing the total pixel area occupied by nuclei by the mean pixel area of one nucleus in the uninfected condition at 12 h post-movie start.

### Replication assay

H1299-E3 cells were seeded at 1 × 10^6^ cells in 20 ml growth medium in a Corning T75 flask 18–20 h pre-infection. Cells were infected with 10 focus forming units of either D614G, D6, or D190. A 1.5 ml of supernatant was collected at input (day 0) and on days 1–3 post-infection with and culture replenished with1.5 ml fresh medium. Samples were sent to an accredited diagnostic laboratory (Molecular Diagnostic Services, Durban, South Africa) to determine SARS-CoV-2 cycle threshold (*Ct*) values where samples were extracted using a guanidine isothiocyanate/magnetic bead-based method with the NucliSense (Biomerieux) extractor of the KingFisher Flex 96 (Thermo Fisher). RT-qPCR was performed using the Seegene Allplex 2019 nCoV assay with the Bio-Rad CFX96 real-time PCR instrument as per the kit instructions. RNase P was used as the internal housekeeping gene to monitor extraction and assay efficiency. The kit targets the E, N, and R genes of SARS-CoV-2. Run calls and interpretation was performed by the Seegene Viewer software. Fold-change was calculated as FC = 2^((mean(^*^Ct^*  ^input)—^*^Ct^*  ^sample)^.

### Detection of infected cell death

H1299-E3 cells were plated at 60,000 cells per well in 6-well plates (Corning) 1 day pre-infection. The next day the cells were infected at 1000 focus-forming units in 1 ml growth media per well. Cell–virus mixtures were incubated for 1 h at 37°C, 5 per cent CO_2_ then an additional 1 ml of growth media was added. Twenty-four hours post-infection, cells were trypsinized (Sigma-Aldrich), collected, and stained with Blue Live/Dead stain as per manufacturer instructions (L34961, ThermosScientific). The samples were then washed in 1 ml PBS^−/−^ and resuspended in Cytofix/Cytoperm (BD Biosciences) for 20 min at 4˚C in the dark. The samples were then stained with 0.5 μg/ml anti-SARS-CoV-2 nucleocapsid-PE (ab283244, Abcam) for 1 h at 4˚C in the dark. Cells were analysed on an Aria Fusion (BD). Data were analyzed using FlowJo and Graphpad Prism 9.4.1 software.

### Staining for cell-surface and total spike in a focus forming assay

H1299-E3 cells were plated in a 96-well plate (Corning) at 20,000 cells per well 1 day pre-infection. Virus stocks were used at 100 focus-forming units per microwell. Cells were infected with 100 μl of the virus for 1 h, then 100 μl of a 1X RPMI 1640 (Sigma-Aldrich, R6504), 1.5 per cent carboxymethylcellulose (Sigma-Aldrich, C4888) overlay was added without removing the inoculum. Cells were fixed at 18 h post-infection using 4 per cent methanol-free formaldehyde (ThermoScientific) for 20 min. For staining of foci, a rabbit anti-SARS-CoV-2 spike monoclonal antibody (mAb BS-R2B12, GenScript A02058) at 0.5 µg/ml or a rabbit anti-SARS-CoV-2 nucleocapsid monoclonal (ab271180 Abcam) at 1 µg/ml were used as the primary detection antibody. Antibody was resuspended in either a permeabilization buffer containing 0.1 per cent saponin (Sigma-Aldrich), 0.1 per cent BSA (Sigma-Aldrich), and 0.05 per cent Tween-20 (Sigma-Aldrich) in PBS^+/+^ or a non-permeabilization buffer containing 0.1 per cent BSA and 0.05 per cent Tween-20 in PBS^+/+^ . Plates were incubated with primary antibody at room temperature for 2 h with shaking, then washed with wash buffer containing 0.05 per cent tween in PBS^+/+^ . Secondary goat anti-rabbit horseradish peroxidase (Abcam ab205718) was added at 1 µg/ml in either permeabilization or non-permeabilization buffers as described earlier and incubated for 2 h at room temperature with shaking. TrueBlue peroxidase substrate (SeraCare 5510–0030) was then added at 50 µl per well and incubated for 15 min at room temperature. Plates were washed in distilled water and then dried for 2 h and imaged in an ImmunoSpot Ultra-V S6-02-6140 Analyzer ELISPOT instrument with BioSpot Professional built-in image analysis (C.T.L). Data were analyzed using Graphpad Prism 9.4.1.

## Supplementary Material

vead075_Supp
