# Supplementary material for: SARS-CoV-2 infection in immunosuppression evolves sub-lineages which independently accumulate neutralization escape mutations
Source: Virus Evol. 2023 Dec 28;10(1):vead075. doi: 10.1093/ve/vead075 (PMC10868398; doi:10.1093/ve/vead075)
Supplement: vead075_Supp [file vead075_supp.zip › Table S1.docx]

Table S1: Summary information for participants infected in the ancestral D614G infection wave

|  | n=9 |
| --- | --- |
| Age (median, IQR) | 49 (45-66) |
| Female | 5 (56%) |
| Days post-infection to sample (median, IQR) | 27 (25-28) |
| People living with HIV | 2 (22%) |
