# Supplementary material for: SARS-CoV-2 infection in immunosuppression evolves sub-lineages which independently accumulate neutralization escape mutations
Source: Virus Evol. 2023 Dec 28;10(1):vead075. doi: 10.1093/ve/vead075 (PMC10868398; doi:10.1093/ve/vead075)
Supplement: vead075_Supp [file vead075_supp.zip › Table S2.docx]

Table S2: Per participant information for participants infected in the ancestral D614G infection wave

| # | Sex | Age range | Sample collection date | Infection  date | Infect. to  sample (days) | HIV Status | Day6 FRNT50 | Day34 FRNT50 | Day71 FRNT50 | Day190 FRNT50 | D614G FRNT50 | Beta FRNT50 | Delta FRNT50 |
| --- | --- | --- | --- | --- | --- | --- | --- | --- | --- | --- | --- | --- | --- |
| 1 | M | 50-59 | Jul 20 | Jun 20 | 25 | - | 1033 | 209 | 596 | 183 | 1177 | 189 | 472 |
| 2 | F | 40-49 | Aug 20 | Jul 20 | 31 | + | 360 | 139 | 166 | 81 | 432 | 95 | 286 |
| 3 | F | 60-69 | Jul 20 | Jul 20 | 28 | - | 954 | 189 | 448 | 251 | 1448 | 291 | 810 |
| 4 | F | 60-69 | Aug 20 | Jul 20 | 27 | - | 310 | 148 | 447 | 415 | 2252 | 326 | 519 |
| 5 | F | 40-49 | Jul 20 | Jun 20 | 24 | - | 952 | 397 | 667 | 287 | 1119 | 179 | 1347 |
| 6 | F | 40-49 | Jul 20 | Jul 20 | 24 | - | 398 | 90 | 244 | 108 | 739 | 281 | 333 |
| 7 | M | 30-39 | Aug 20 | Jul 20 | 36 | - | 258 | 65 | 106 | 137 | 271 | 164 | 275 |
| 8 | M | 40-49 | Aug 20 | Jul 20 | 28 | + | 465 | 119 | 372 | 107 | 954 | 132 | 437 |
| 9 | M | 60-69 | Sep 20 | Aug 20 | 26 | - | 779 | 342 | 415 | 265 | 913 | 456 | 660 |

Infection date is by date of first available positive qPCR test. All participants living with HIV were HIV suppressed (HIV viral load <200 copies/mL).
