# Supplementary material for: SARS-CoV-2 infection in immunosuppression evolves sub-lineages which independently accumulate neutralization escape mutations
Source: Virus Evol. 2023 Dec 28;10(1):vead075. doi: 10.1093/ve/vead075 (PMC10868398; doi:10.1093/ve/vead075)
Supplement: vead075_Supp [file vead075_supp.zip › Table S4.docx]

Table S4: Per participant information for participants infected in the Beta infection wave

| # | Sex | Age range | Sample collection date | Infection  date | Infect. to  sample (days) | HIV Status | Day6 FRNT50 | Day34 FRNT50 | Day71 FRNT50 | Day190 FRNT50 | D614G FRNT50 | Beta FRNT50 | Delta FRNT50 |
| --- | --- | --- | --- | --- | --- | --- | --- | --- | --- | --- | --- | --- | --- |
| 1 | M | 50-59 | Feb 21 | Jan 21 | 28 | - | 944 | 798 | 510 | 1114 | 410 | 1658 | 279 |
| 2 | F | 30-39 | Feb 21 | Jan 21 | 27 | - | 550 | 298 | 473 | 506 | 288 | 514 | 315 |
| 3 | M | 60-69 | Feb 21 | Jan 21 | 18 | - | 498 | 440 | 189 | 499 | 232 | 697 | 274 |
| 4 | F | 60-69 | Feb 21 | Jan 21 | 29 | - | 252 | 307 | 225 | 652 | 158 | 1014 | 99 |
| 5 | F | 30-39 | Feb 21 | Jan 21 | 31 | +^#^ | 93 | 82 | 56 | 284 | 58 | 240 | 94 |
| 6 | M | 70-79 | Feb 21 | Feb 21 | 20 | - | 1709 | 1154 | 817 | 1517 | 464 | 3398 | 1213 |
| 7 | F | 50-62 | Mar 21 | Feb 21 | 22 | - | 302 | 181 | 340 | 433 | 186 | 1212 | 315 |
| 8 | F | 50-59 | Feb 21 | Jan 21 | 35 | - | 3605 | 3623 | 3251 | 2886 | 2392 | 3612 | 866 |
| 9 | M | 50-59 | May 21 | Jan 21 | 120 | + | 2095 | 1522 | 1467 | 2047 | 1795 | 2856 | 1279 |
| 10 | M | 60-69 | Mar 21 | Mar 21 | 21 | - | 1052 | 700 | 479 | 865 | 199 | 1618 | 338 |
| 11 | F | 70-79 | Feb 21 | Jan 21 | 48 | + | 788 | 355 | 619 | 946 | 358 | 726 | 370 |

Infection date is by date of first available positive qPCR test. All participants living with HIV were HIV suppressed (HIV viral load <200 copies/mL) except for ^#^, where HIV viral load = 1166.
