# Supplementary material for: SARS-CoV-2 infection in immunosuppression evolves sub-lineages which independently accumulate neutralization escape mutations
Source: Virus Evol. 2023 Dec 28;10(1):vead075. doi: 10.1093/ve/vead075 (PMC10868398; doi:10.1093/ve/vead075)
Supplement: vead075_Supp [file vead075_supp.zip › Table S6.docx]

Table S6: Per participant information for participants infected in the Delta infection wave

| # | Sex | Age range | Sample collection date | Infection  date | Infect. to  sample (days) | HIV Status | Day6 FRNT50 | Day34 FRNT50 | Day71 FRNT50 | Day190 FRNT50 | D614G FRNT50 | Beta FRNT50 | Delta FRNT50 |
| --- | --- | --- | --- | --- | --- | --- | --- | --- | --- | --- | --- | --- | --- |
| 1 | F | 40-49 | Aug 21 | Jul 21 | 24 | - | 2955 | 555 | 1025 | 227 | 521 | 276 | 5979 |
| 2 | M | 40-49 | Aug 21 | Jul 21 | 24 | +^#^ | 686 | 169 | 543 | 153 | 677 | 147 | 2709 |
| 3 | M | 50-59 | Aug 21 | Jul 21 | 23* | + | 2462 | 684 | 1061 | 323 | 711 | 299 | 19554 |
| 4 | M | 40-49 | Aug 21 | Aug 21 | 13 | + | 715 | 129 | 299 | 70 | 138 | 81 | 3955 |
| 5 | M | 40-49 | Aug 21 | Jul 21 | 27 | - | 2133 | 715 | 737 | 278 | 841 | 446 | 8427 |
| 6 | M | 50-59 | Jul 21 | Jun 21 | 37 | - | 2055 | 537 | 963 | 226 | 548 | 340 | 8101 |
| 7 | M | 40-49 | Jul 21 | Jul 21 | 22 | - | 898 | 269 | 1789 | 251 | 3429 | 135 | 5453 |
| 8 | M | 30-39 | Jul 21 | Jul 21 | 22 | - | 202 | 94 | 290 | 70 | 162 | 73 | 1565 |
| 9 | F | 50-59 | Jul 21 | Jul 21 | 23 | - | 2674 | 1115 | 1771 | 649 | 1069 | 570 | 12457 |

Infection date is by date of first available positive qPCR test. All participants living with HIV were HIV suppressed (HIV viral load <200 copies/mL) except for ^#^, where HIV viral load = 34261. *: Vaccinated prior to sample collection.
