# Supplementary material for: SARS-CoV-2 infection in immunosuppression evolves sub-lineages which independently accumulate neutralization escape mutations
Source: Virus Evol. 2023 Dec 28;10(1):vead075. doi: 10.1093/ve/vead075 (PMC10868398; doi:10.1093/ve/vead075)
Supplement: vead075_Supp [file vead075_supp.zip › Table S7.docx]

Table S7: Sequences of isolated SARS-CoV-2 used in this study

| Viral isolate | GISAID accession | PANGO |
| --- | --- | --- |
| D614G.1 | EPI_ISL_602626.1 | B.1 |
| D614G.2 | EPI_ISL_602622 | B.1.1.117 |
| Beta | EPI_ISL_678615 | B.1.351 |
| Delta | EPI_ISL_3118687 | B.1.617.2 |
| BA.1 | EPI_ISL_7886688 | BA.1 |
| BA.5 | EPI_ISL_12268493.2 | BA.5.2 |
| 0027-D6-R682W | EPI_ISL_15541746 | B.1.1.273 |
| 0027-D6 | EPI_ISL_18050145 | B.1.1.273 |
| 0027-D20 | EPI_ISL_15541747 | B.1.1.273 |
| 0027-D34 | EPI_ISL_15541748 | B.1.1.273 |
| 0027-D71 | EPI_ISL_15541749 | B.1.1.273 |
| 0027-D106 | EPI_ISL_15541750 | B.1.1.273 |
| 0027-D190 | EPI_ISL_15541751 | B.1.1.273 |
