# Supplementary figures and images for: SARS-CoV-2 infection in immunosuppression evolves sub-lineages which independently accumulate neutralization escape mutations
Source: Virus Evol. 2023 Dec 28;10(1):vead075. doi: 10.1093/ve/vead075 (PMC10868398; doi:10.1093/ve/vead075)

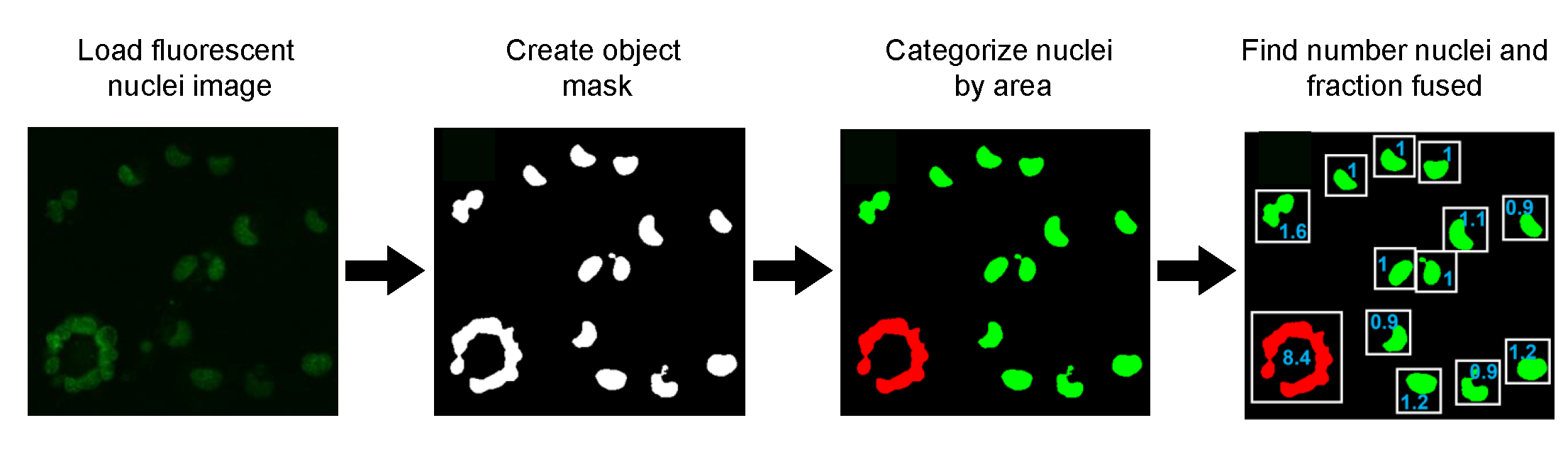

Supplement: vead075_Supp [file vead075_supp.zip › FigS1_300dpi.tif]

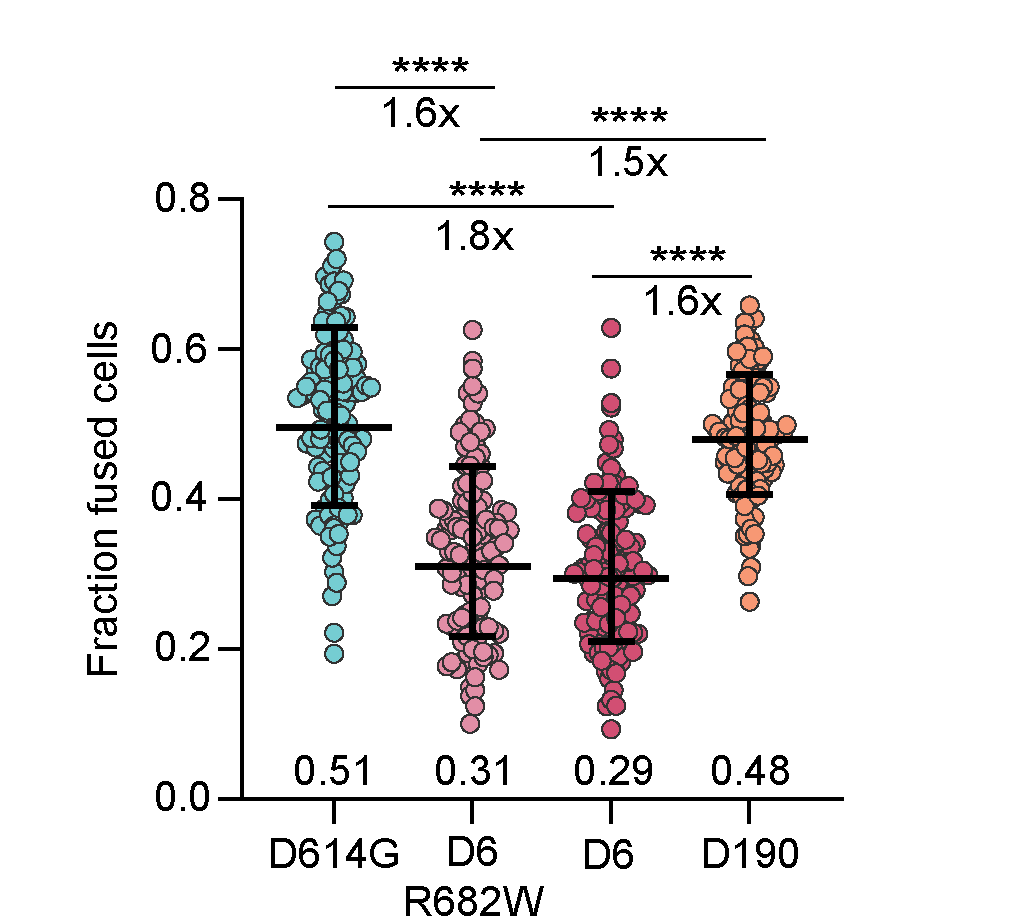

Supplement: vead075_Supp [file vead075_supp.zip › FigS2_300dpi.tif]

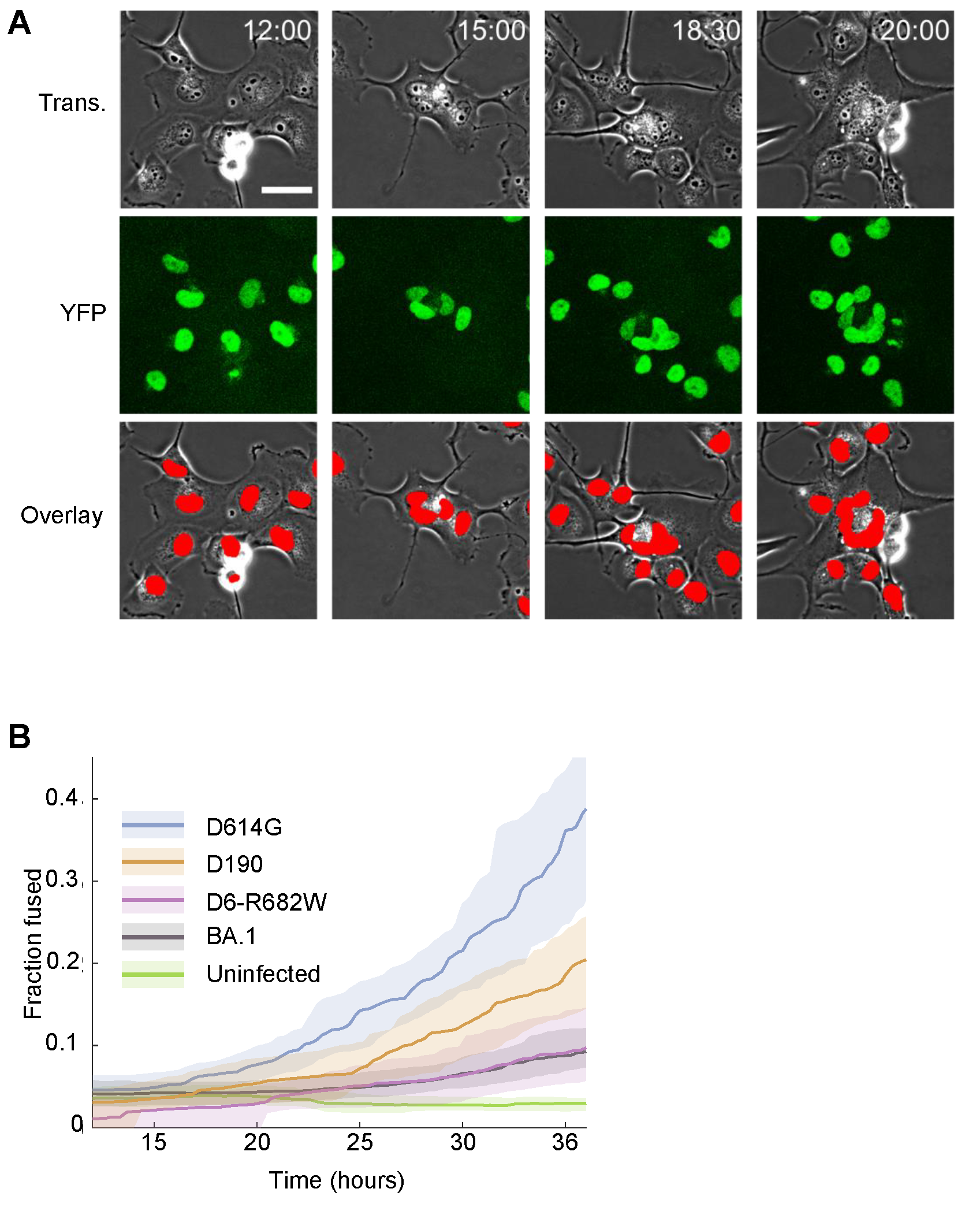

Supplement: vead075_Supp [file vead075_supp.zip › FigS3_300dpi.tif]

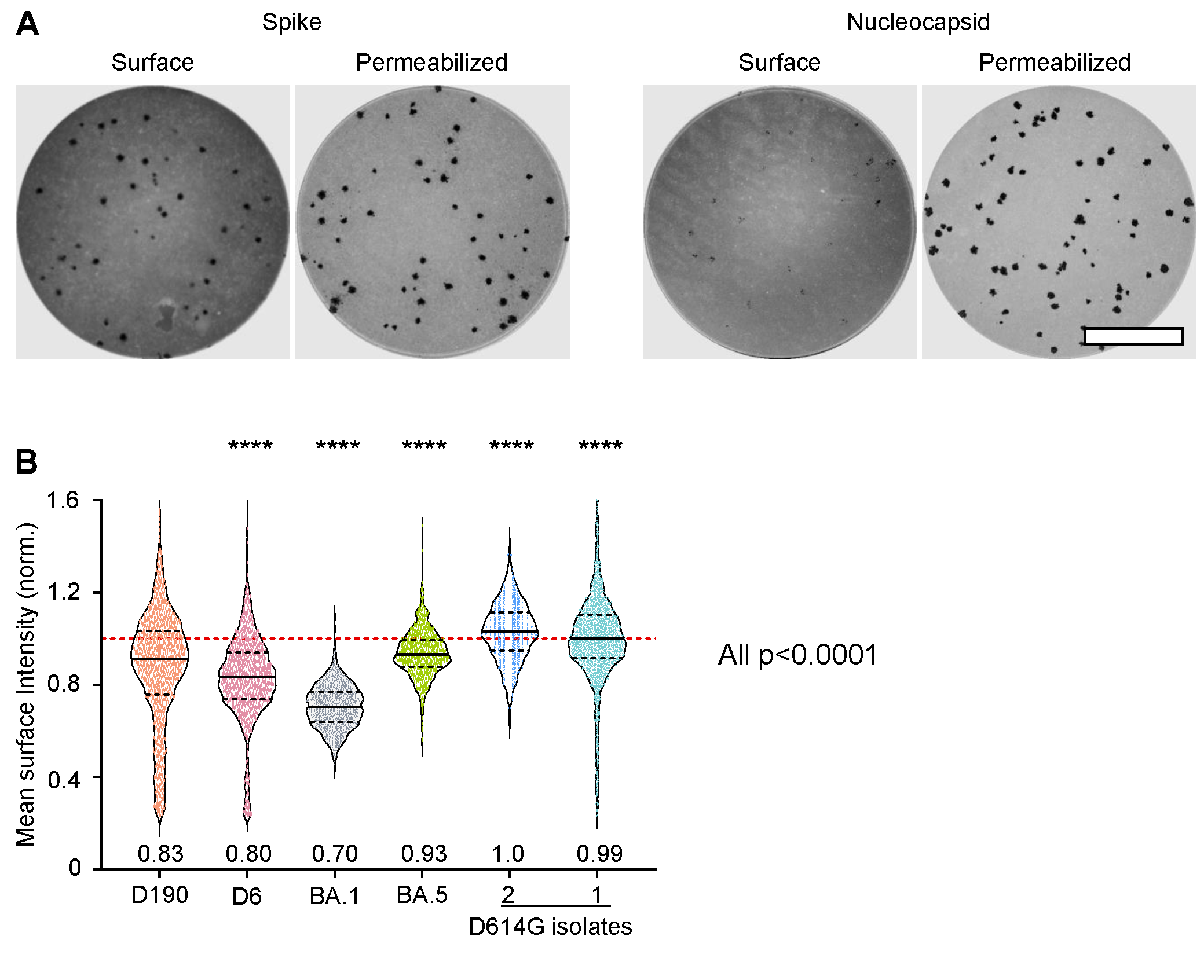

Supplement: vead075_Supp [file vead075_supp.zip › FigS4_300dpi.tif]
